# Symptomatic Fracture of the Subhallucal Interphalangeal Sesamoid Bone: A Case Report

**DOI:** 10.7759/cureus.79618

**Published:** 2025-02-25

**Authors:** Devin J Farrell, Catherine Riché

**Affiliations:** 1 Medicine, William Carey University College of Osteopathic Medicine, Hattiesburg, USA; 2 Orthopedic Surgery/Foot and Ankle, Baton Rouge Orthopedic Clinic, Baton Rouge, USA

**Keywords:** interphalangeal joint, interphalangeal sesamoid, os interphalangeus, sesamoid, sesamoid fracture, subhallucal

## Abstract

The hallucal interphalangeal sesamoid bone can often be misdiagnosed when fractured and show presenting symptoms of forefoot pain. Primary presentation is asymptomatic but can become symptomatic in cases of trauma, overuse, or pressure to the area. However, even when symptomatic, misdiagnosis can often occur and lead to improper management that can exacerbate or prolong symptoms. This case is unique in revealing a rare anatomical variation and shows the proper approach to treatment and management from a prior misdiagnosis of right big toe pain without indication of a fracture.

## Introduction

Forefoot pain is a common occurrence frequently encountered in practice. Interphalangeal sesamoid pathology causative of forefoot pain is often misdiagnosed and overlooked. However, they play a crucial role in weight bearing the foot and hallux. Sesamoid formation within the interphalangeal joint (IPJ) varies in occurrence with incidences ranging from 2% to 13% and an average of 27.5% [1-4]. Symptomatic issues can occur due to overuse, trauma, or pressure on the area, which can affect the flexor hallucis longus (FHL) at times [5]. Additionally, biomechanics within the foot may be altered by the presence of an interphalangeal sesamoid or accessory ossicles within the joint and cause deformities [2,6,7]. As a result, this may be perceived as a simple big toe sprain on presentation. Many physicians misdiagnose the injury of the hallux, often leading to delay of proper treatment and causing further fatal implications such as osteonecrosis of the sesamoid bone or sesamoiditis [4,8]. Earlier findings of fracturing, dislocation, or other modes of trauma to the sesamoid can help prevent misdiagnosis and progression of symptoms.

This case report aims to provide valuable insight into understanding the proper management of big toe pain due to a subhallucal interphalangeal sesamoid fracture and review the literature on the anatomical variations' pathologies.

## Case presentation

A 22-year-old male, with no prior past medical history, initially presented to the orthopedic after-hours clinic two days after an incident where he ran into and collided with another player while playing soccer. Following the encounter, he was diagnosed with a right big toe sprain with no fractures noted and was told to ice and elevate the foot. However, despite following the treatment regimen, he still experienced pain, which progressively worsened over the past two weeks and required re-evaluation at our outpatient clinic. He described it as a lingering sharp pain located at the IPJ of the right great toe.

**Figure 1:**
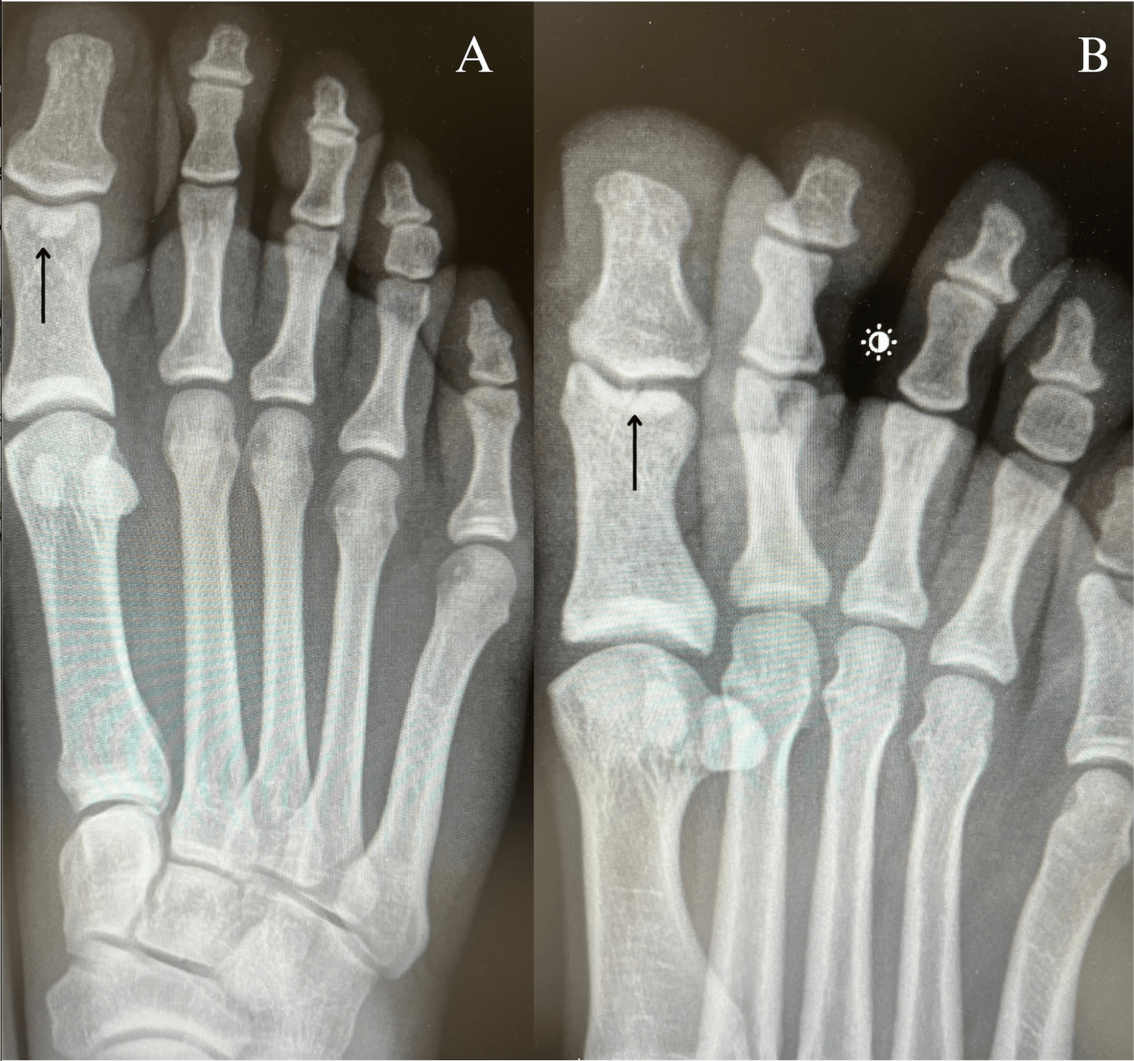
Plain anteroposterior (A) and oblique radiographs (B) of the right foot confirm the presence of a fracture in the interphalangeal sesamoid bone at the plantar aspect of the foot within the distal interphalangeal joint of the great toe (black arrows)

On physical exam, there was swelling at the IPJ with a non-radiating moderate sharp tenderness to palpation located at the plantar aspect of the IPJ. The patient was not limited in passive and active range of motion but experienced pain in movement. Resisted extension and flexion of the IPJ elicited tenderness on presentation. No sensory or motor deficits were found. Plain radiographs of the right foot were ordered and revealed a non-displaced avulsion fracture of the interphalangeal sesamoid bone in the right IPJ due to the FHL tendon (Figure 1). Subsequently, after diagnosis, the patient was advised to wear a short boot and remain non-weight bearing on a knee scooter and to follow up in four weeks for X-rays and re-evaluation.

**Figure 2:**
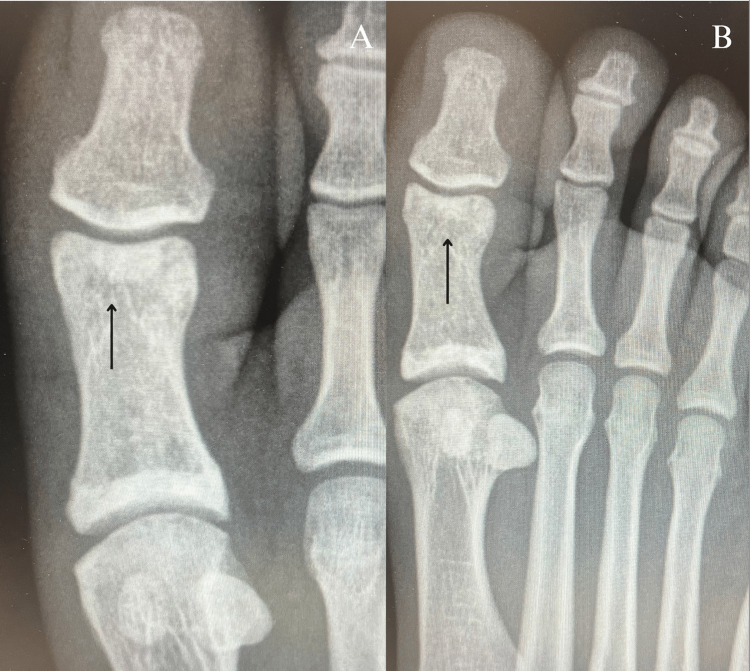
Plain anteroposterior (A) and oblique radiographs (B) of the right foot taken nine weeks and three days from the initial injury show complete ossification of the sesamoid bone fracture at the distal interphalangeal joint of the great toe (black arrows)

The patient’s follow-up radiography after four weeks showed healing of the sesamoid bone, along with resolution of initial symptoms. The patient has since transitioned to weight-bearing in the short boot for three days and then to shoes. Imaging and evaluation of the foot nine weeks after the initial injury showed complete resolution of initial symptoms and complete ossification of the sesamoid bone fracture (Figure 2). The patient describes no current pain at all, with the only complaint being swelling occurring intermittently at the joint area when on his feet. He also states that movement in the joint is improved to the full range of motion without pain, with the exception of only mild pain in flexion of the distal IPJ of the big toe due to trauma.

## Discussion

This case demonstrates the benefits of understanding the underlying causes of great toe pain and provides insight into anatomical anomalies that can occur in the general population, often leading to a misdiagnosis. The incidence rate of the interphalangeal sesamoid of the great toe is 2-13%, with an average of 27.5% [1-4]. In one retrospective radiograph case, it was noted that interphalangeal sesamoids appeared in 13 of the 1,651 cases (0.78%) [9]. Ossicle formation can vary in positioning between plantar, dorsal, or within the IPJ. In our case, ossification of the sesamoid occurred in the plantar aspect of the IPJ. Proper diagnosis and understanding of sesamoid pathology can be beneficial for preventing unnecessary or harmful planning, which can exacerbate symptoms.

In the presence of multiple IPJ sesamoids, irritation of the FHL tendon can occur due to limitation in mobility and alteration of biomechanics [6]. Dislocation may also occur secondarily to forceful hyperextension of the great toe, causing separation of the plantar plate from the proximal phalanx and ultimately disrupting the joint capsule [2,10]. Meanwhile, dislocation is an overt cause of forefoot pain, fractures, avascular necrosis, and sesamoiditis of the bone can be misdiagnosed and should be made aware to clinicians when evaluating big toe pain [4,5,8]. In our case, a previous misdiagnosis of a right big toe sprain overshadowed the sesamoid fracture and led to improper management with prolongation of symptoms. In atraumatic scenarios, one must be mindful of the possibility of patients potentially developing hallux valgus interphalangeus (HVIP) due to accessory ossicles progressively altering the biomechanics of the big toe [7]. Other studies have shown that plantar keratosis can form and can also contribute to altering the biomechanics and increased friction of the foot during hyperextension of IPJ [1,11]. In our case, close monitoring of plain radiography helped diagnose and manage a pathological subhallux sesamoid fracture that had initially gone unnoticed. To our knowledge, no other cases have been recorded for a pathological fracture of the subhallux interphalangeal sesamoid bone.

Diagnostic imaging varies to confirm the cause of forefoot pain. Initial imaging, following a physical examination of the affected area, should always be done with plain radiography. The usage of ultrasound, however, has been argued as a more sensitive and non-invasive first-line diagnostic tool to assess for the presence of interphalangeal ossicles, as well as any associated bursitis or FHL tenosynovitis [11,12]. Prior knowledge of sesamoid pathology and anatomical variation allows for a quick and concise diagnosis for imaging evaluation without delay of treatment. In the absence of dislocation, fracturing, and other deformities, MRI allows for an in-depth review of potential osteonecrosis or sesamoiditis of the bone. However, early stages of sesamoiditis and osteonecrosis can give similar imaging characteristics of bone marrow edema, which lacks effective differentiation on MRI [4]. Further differentiation between the two should, therefore, be with CT imaging [3,4]. In the case of Kumar et al., CT and MRI must be used to differentiate the poorly corticated margins of irregular bony fragments in fractures from the smooth margins in sesamoiditis [8].

The successful treatment measures implemented in this case involved booting the patient and keeping the patient non-weight-bearing while taking oral nonsteroidal anti-inflammatory drugs. Ultrasound-guided injections with long-acting anesthetic agents mixed with steroids have shown good results in alleviating inflammation between the sesamoid and FHL tendon [4,5,12]. The best approach would be a medial plantar approach or a horizontal approach for injection [12]. In the absence of improvement in symptoms, minimally invasive surgical resection of the sesamoid bone is indicated to reduce complications and prevent scar tissue formation [1,5,6]. Ultrasound-guided surgery may be favored over open surgery for patients with higher morbidities due to the “ultra minimally invasive” aspect of the surgery. Allowing for minimal side effects and fibrosis of tissue due to a smaller incision [11]. The clinician must decide the best surgical approach based on patient history and compliance.

## Conclusions

This case highlights a rare pathological fracture of a variation in sesamoid formation, which should be accounted for when providing proper diagnostic assessments and treatment plans for forefoot pain. Misdiagnosis of the pathology will lead to worsening symptoms of the fracture that can progress to osteonecrosis, tendinitis, or sesamoiditis. Even asymptomatically, one must be wary of interphalangeal sesamoids or additional ossicles in the IPJ for potential causes of pathology due to alteration of biomechanics in the foot. Therefore, clinicians must be knowledgeable of sesamoid formation and variation within the IPJ to differentiate from common causes of forefoot pain for precise and quick treatment. While treatment varies on diagnosis, it is recommended to start conservatively with alleviation of pressure to the forefoot and reserve surgical consideration for when all other modalities do not resolve symptoms. Our recommendation in conservative treatment would be to keep the patient non-weight bearing and booted to alleviate pressure at the IPJ and promote proper healing of sesamoid fractures. This, in turn, will allow for a rapid return in physical activities and quality of life. To the best of our knowledge, this is the first occurrence of literature on a pathologic fracture of the interphalangeal sesamoid in the big toe. Therefore, comparative data on healing time, symptoms, and misdiagnosis of the fracture are limited. Further studies would need to be conducted to assess the average healing time of interphalangeal sesamoid fracture, misdiagnosis rate, and common presenting symptoms to provide further knowledge of the pathology.
